# A Wide Linear Range Eddy Current Displacement Sensor Equipped with Dual-Coil Probe Applied in the Magnetic Suspension Flywheel

**DOI:** 10.3390/s120810693

**Published:** 2012-08-06

**Authors:** Jiancheng Fang, Tong Wen

**Affiliations:** Science and Technology on Inertial Laboratory, School of Instrumentation Science and Opto-Electronics Engineering, Beijing University of Aeronautics and Astronautics, Beijing 100083, China; E-Mail: fangjiancheng@buaa.edu.cn

**Keywords:** eddy current displacement sensor, dual-coil probe, magnetic suspension flywheel, FEM analysis

## Abstract

The Eddy Current Displacement Sensor (ECDS) is widely used in the Magnetic Suspension Flywheel (MSFW) to measure the tiny clearance between the rotor and the magnetic bearings. The linear range of the ECDS is determined by the diameter of its probe coil. Wide clearances must be measured in some new MSFWs recently designed for the different space missions, but the coil diameter is limited by some restrictions. In this paper, a multi-channel ECDS equipped with dual-coil probes is proposed to extend the linear range to satisfy the demands of such MSFWs. In order to determine the best configuration of the dual-coil probe, the quality factors of the potential types of the dual-coil probes, the induced eddy current and the magnetic intensity on the surface of the measuring object are compared with those of the conventional single-coil probe. The linear range of the ECDS equipped with the selected dual-coil probe is extended from 1.1 mm to 2.4 mm under the restrictions without adding any cost for additional compensation circuits or expensive coil materials. The effectiveness of the linear range extension ability and the dynamic response of the designed ECDS are confirmed by the testing and the applications in the MSFW.

## 1. Introduction

The magnetic suspension flywheel (MSFW), whose rotor is suspended by magnetic bearings (MB) is regarded as the most promising attitude actuator for ultra-high precision satellites [1,2]. Because of the unstable nature of the actively controlled MB, a number of displacement sensors are used in the MSFW to acquire displacement information about the rotor for the feedback control of the MB. The eddy current displacement sensor (ECDS) is widely used in the MSFW thanks to its advantages such as high sensibility and reliability, compact detecting circuit, wide operating temperature range and so on [3,4]. The clearance between the rotor and the MB to be measured of the traditional MSFW is usually less than 1 mm. In recent years, a number of new kinds of MSFWs were designed to satisfy the different demands of the satellites for different space missions. For instance, the Gimbaling-MSFW (G-MSFW) was invented to generate giant torque instantaneously by tilting the spinning rotor [5], the active-passive hybrid MSFW (APH-MSFW), whose rotor is suspended both by the active MBs and passive MBs, was designed to save the power consumption in some degrees of freedom (DOF) [6,7]. The similarity of the two kinds MSFW is that the clearances between the rotor and MBs in the axial translation and radial rotations are much longer than in the ordinary MSFW, meaning that a multi-channel ECDS with larger linear range in those DOFs is needed both by the G-MSFW and APH-MSFW.

The linear range of the ECDS could be extended by increasing the diameter of the probe coil [8]. Because the diameter is limited by the mechanical-structural features of such MSFWs, this method could not to be applied to extend the linear range. Because of the lack of the application experience in the space, new coil materials such as the magnetoplated wire mentioned in [9,10] are not suitable for the MSFW with space missions. Because of the strict restrictions on the volume and mass of the MSFW, all probes of the ECDS are mounted in one frame and the detection circuits of these probes are integrated on one printed circuit broad (PCB), as a result, there is no enough room for any additional compensation circuits which could extend the linear range of the ECDS easily. The linear range of the ECDS is determined by the quality factor (*Q*-factor) of the probe coil and the induced eddy current density on the surface of the measuring object [11]. In order to satisfy the requirement of the large gap measurement of the MSFW with the three limitations mentioned above, in this paper a dual-coil probe which could extend the linear range of the ECDS is introduced without any additional cost of more compensation circuits or expensive coil materials. The diameter of the coils used in the designed ECDS is kept the same as in the conventional single-coil ECDS.

In this paper, the following points concerning the ECDS equipped with the dual-coil probe are discussed:

1. The physical phenomena of the ECDS.
2. The configuration and connection modes of the pair of coils.
3. The *Q*-factors of the four dual-coil probe candidates and the single coil probe.
4. The distributions of the induced eddy current density and magnetic intensity on the surface of the measuring object.
5. The bandwidth of the dual-coil probe ECDS.

Finally, the ability of the proposed dual-coil probe to extend the linear range of the ECDS is confirmed by the test results and applications in the MSFW.

## 2. Design of the Dual-Coil Probe ECDS

### 2.1. Configuration of the Multi-Channel ECDS

**Figure 1.**
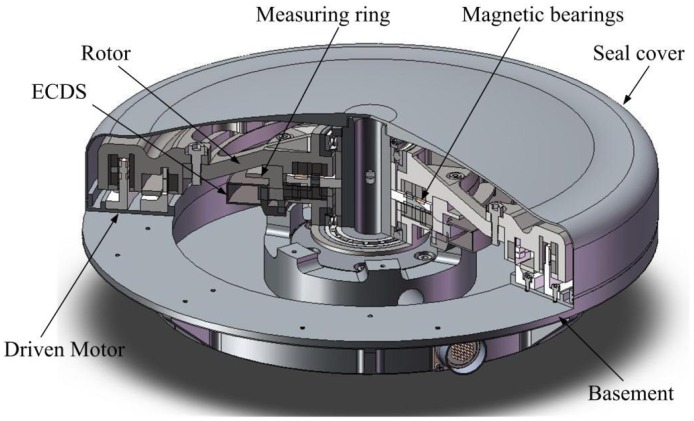
The sketch of the MSFW whose rotor is suspended by the magnetic bearings and monitored by the multi-channel ECDS.

Figure 1 is a sketch of the MSFW whose rotor is suspended by the MBs. The MSFW consists of the rotor, magnetic bearings, ECDS, measuring ring of the ECDS, driven motor, seal cover and basement. The displacement and attitude information of the rotor could be calculated according to the measured gap lengths between the probes of the multi-channel ECDS mounted on the basement and the measuring ring fixed in the rotor.

**Figure 2.**
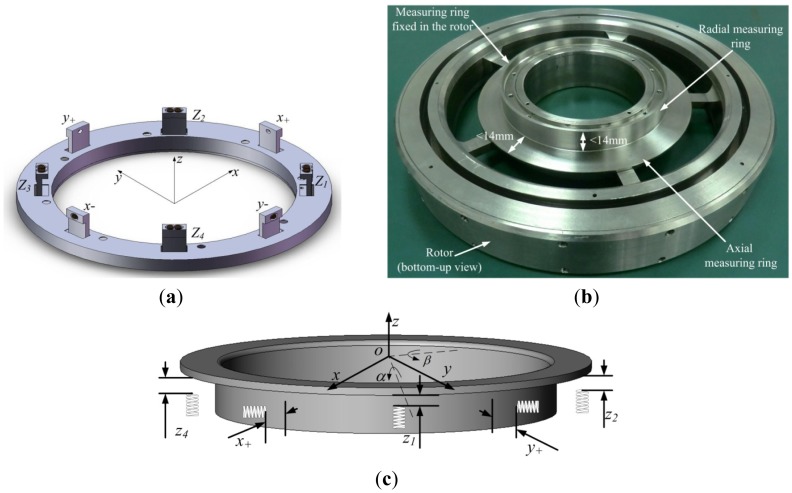
The 9-channel ECDS applied in the MSFW (**a**) The probe configuration of the 9-channel ECDS; (**b**) The measuring ring assembled in the rotor; (**c**) The relations between the generalized displacements of the rotor and the clearance lengths measured by the 9-channel ECDS.

The configuration of the 9-channel ECDS is described in Figure 2(a). The details of the measuring ring that is fixed in and rotates with rotor are described in Figure 2(b). Figure 2(c) shows the relations between the generalized displacements of the rotor and the gap lengths measured by the ECDS. The radial translation displacements [*x y*] of the rotor could be calculated from the four radial probes *x*_+_, *x*_−_, *y*_+_ and *y*_−_, the axial translation *z* and the two axial rotation displacements [*α β*] of the rotor could be calculated from the four axial probes *z_1_*–*z_4_*. The *z_0_* probe is fixed in the ECDS and introduced to compensation the temperature drift of the *z_1_*–*z_4_* probes. The transfer matrix from the outputs of the ECDS [*x*_+_ *x*_−_ *y*_+_ *y*_−_ *z_0_ z_1_ z_2_ z_3_ z_4_*] to the displacements [*x y z α β*] is described in Equation (1):

(1) $$\begin{array}{l} \left\{ \begin{array}{l} x=x_{+}-x_{-}\\ y=y_{+}-y_{-} \end{array}\right.,\{\begin{array}{c} z={z^{\prime }}_{1}+{z^{\prime }}_{2}+{z^{\prime }}_{3}+{z^{\prime }}_{4}\\ \alpha =\left({z^{\prime }}_{1}-{z^{\prime }}_{3}\right){/D}_{s}\\ \beta =\left({z^{\prime }}_{2}-{z^{\prime }}_{4}\right){/D}_{s} \end{array}\\ {z^{\prime }}_{i}=z_{i}-z_{0},\left(i=1\cdots 4\right) \end{array}$$

The linear range of ECDS is about 1/5 (0.5% degree of linearity) of the diameter of the probe coil without linear compensation [12]. The width of the measuring object is required to be triple the diameter of the coil, at least. As shown in Figure 2(b), the diameter of the used probe coil is 4.7 mm, limited by the width of the measuring ring of less than 14 mm. The linear range of the single-coil ECDS is about 1 mm. The linear range of the conventional single coil ECDS thus meets the demands of the ordinary MSFW, but for the G-MSFW and APH-MSFW, the gap-lengths that need to be measured in the axial direction *z_1_*–*z_4_* are more than 2.2 mm and much more than the linear range of conventional ECDS. The much more important is that there is no room for a linear compensation circuit as the area of the PCB is limited by the volume of the ECDS frame. In order to extend the linear range by increasing the *Q*-factor of the probe coil and the induced eddy current density on the surface of the measuring ring, a dual-coil probe ECDS is introduced herein.

### 2.2. The Basic Principle of the ECDS

An ECDS consist of three major components: the oscillator, which generates a radio frequency signal, the probe, which radiates the signal through the coil in the probe, and the modulating circuit, which converts the returned signal into usable form. When the measuring ring is placed near the probe coil, an eddy current will be induced on the surface of the measuring ring. The resulting loss of strength in the returned signal is measured and converted by the detecting circuit into a voltage signal, which is proportional to the gap length between the probe coil and the measuring object [13].

**Figure 3.**
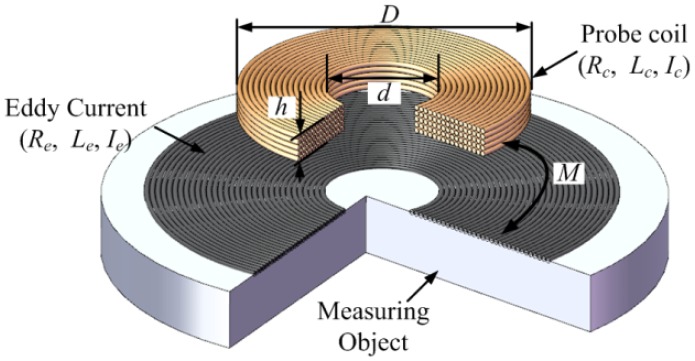
The schematic drawing of the basic principle of the ECDS.

As shown in Figure 3, *R_c_* and *L_c_* are the resistance and inductance of the coil when it is far away from the measuring object, respectively. *R_e_* and *L_e_* are the equivalent resistance and inductance of induced eddy current circle on the surface of the measuring object when the coil is close to the measuring object, and *x* is the distance to be measured between the coil and the measuring object. The variable *M* is introduced to describe the coupling between the coil and the measuring object. When the coil is close to the measuring object, the resistance and inductance of the coil *R*(*x*) and *L*(*x*) become:

(2) $$\text{R}\left(x\right){=\text{R}}_{c}+R_{e}\frac{{\left(2\pi f\right)}^{2}M^{2}}{R_{e}^{2}+{\left(2\pi fL_{e}\right)}^{2}},L\left(x\right){=L}_{c}-L_{e}\frac{{\left(2\pi f\right)}^{2}M^{2}}{R_{e}^{2}+{\left(2\pi fL_{e}\right)}^{2}}$$

**Figure 4.**
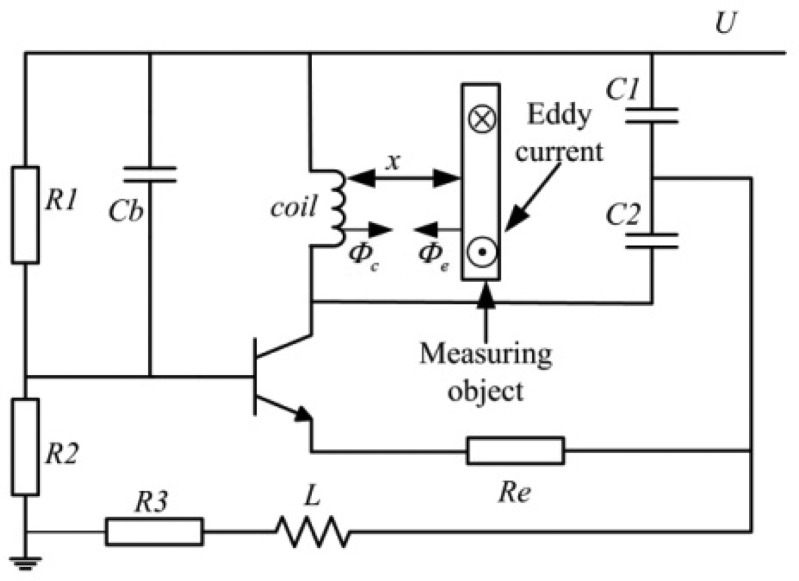
The self-oscillator Clapp circuit used in the ECDS.

In order to generate a high-frequency oscillation signal to drive the coil, the coil is connected in a self-oscillator Clapp circuit, as shown in Figure 4. When the measuring ring is close to the probe, the eddy-current will be induced in its surface based on the Faraday's electromagnetic induction principle.

The magnetic field produced by the eddy-current will affect the original magnetic field by the probe coil current. As a result, the eddy-current affects the inductance and equivalent impedance of the probe coil. The frequency of the resonance, when the measuring object is close to the coil, is described in Equation (3):

(3) $$f\left(x\right)=1/2\pi \sqrt{\frac{1}{L\left(x\right)}\left(\frac{C_{1}C_{2}}{C_{1}{+C}_{2}}\right)}$$

As shown in Figure 4, the magnetic flux *Φ_c_* is generated by the resonance driving current *I_c_* of the coil. The magnetic flux *Φ_e_* is generated by the eddy current *I_e_* in the measuring object, so *I_e_* changes in accordance with the displacement and the *Q*-factor of the coil changes as a result. The *Q*-factor of the coil converts the flux change in the coil into the amplitude and frequency through the self-oscillator circuit. The change of the amplitude could be transferred to a DC voltage as the output signal of the ECDS by the subsequent detecting circuit. The *Q*-factor of the coil denoted as *Q*(*x*) and influenced by the distance *x* is:

(4) $$Q\left(x\right)=\frac{2\pi fL\left(x\right)}{R\left(x\right)}=Q_{\infty }\left[1-\frac{L_{e}}{L_{c}}\left(\frac{{\left(2\pi f\right)}^{2}M^{2}}{R_{e}^{2}+{\left(2\pi f\right)}^{2}L_{e}^{2}}\right)\right]/\left[1+\frac{R_{e}}{R_{c}}\left(\frac{{\left(2\pi f\right)}^{2}M^{2}}{R_{e}^{2}+{\left(2\pi f\right)}^{2}L_{e}^{2}}\right)\right]$$

where *Q_∞_* is the *Q*-factor of the probe coil while the measuring object is far away from the probe.

### 2.3. The Design of the Dual-Coil ECDS

**Figure 5.**
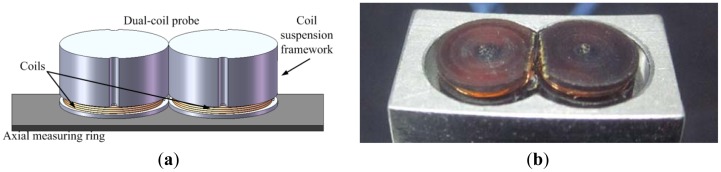
The sketch of the dual-coil ECDS. (**a**) Diagrammatic sketch of the dual-coil probe and the measuring ring; (**b**) Photo of the dual-coil probe.

In order to extend the linear range of the ECDS, not only the *Q*-factor of the probe coil but also the coupling between the probe coil and the eddy current circle should be strengthened. As the diameter of the measuring ring used in the MSFW is much larger than the diameter of the coil, the measuring ring could be regarded as a trap for the probe coil. The details of the measuring ring are described in Figure 2(b). A dual-coil probe is designed by using two identical coils that work together to radiate oscillation signal to enhance the induced eddy current on the surface of the measuring ring. Figure 5 shows the structure of the proposed dual-coil ECDS. Two connected coils are located side by side along the circle of the measuring ring. The detecting circuits, the diameter of the coil and the width of the measuring ring keep the same as the single coil ECDS.

**Figure 6.**
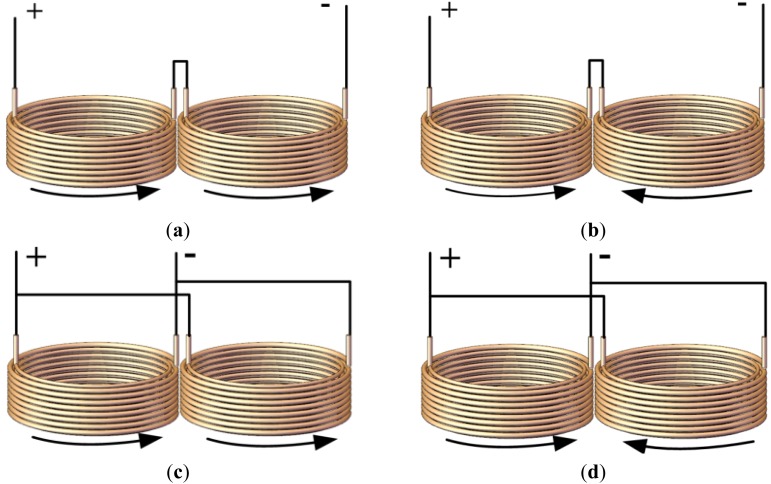
The configuration types of the dual-coil probes. (**a**) Type A. serial connected, same current direction; (**b**) Type B. serial connected, opposite current direction; (**c**) Type C. parallel connected, same current direction; (**d**) Type D. parallel connected, opposite current direction.

According to the connection mode and the instantaneous directions of the exciting currents in the two coils, the dual-coil probes could be classified into four configuration types. They are serial connected with the same current direction (Type A), serial connected with opposite current direction (Type B), parallel connected with same current direction (Type C) and parallel connected with opposite current direction (Type D). Figure 6 shows the details of the four candidates of the dual-coil probes.

## 3. The *Q*-Factors of the Dual-Coil Probes

**Figure 7.**
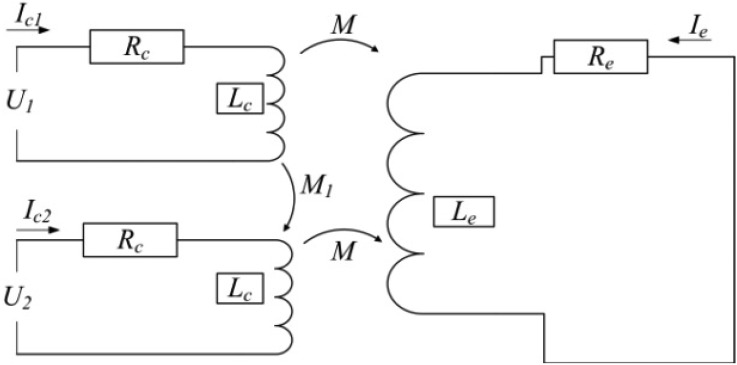
The equivalent circuit of the dual-coil probe and the measuring object.

The *Q*-factor is essential for the linear range of the ECDS. Figure 7 shows the equivalent calculation circuit of the dual-coil probe and the measuring object. According to the equivalent circuit, the *Q*-factors of the dual-coil probes could be calculated and compared with the ordinary single coil probe. The variable *M_1_* is introduced to indicate the coupling between the two connected coils. The sign of the *M_1_* is determined by the current direction in the two coils.

**Figure 8.**
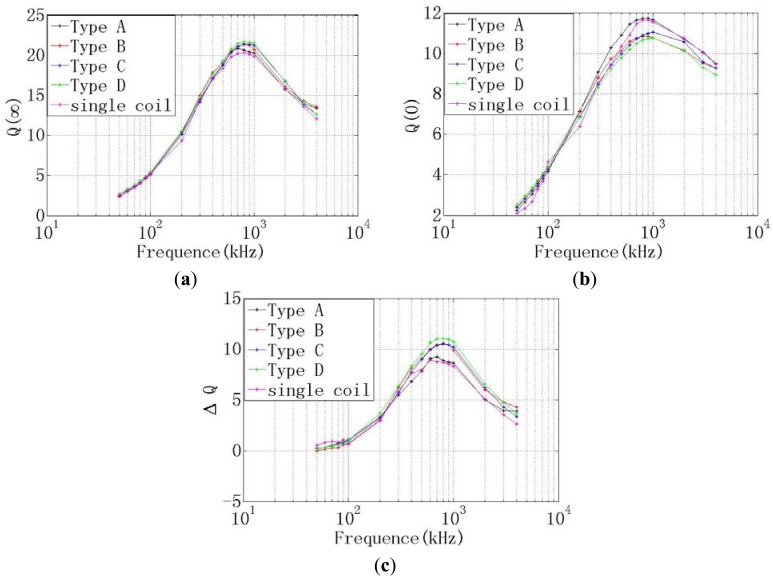
Comparison of the *Q*-factors of the four dual-coil probes and the single-coil probe (**a**) *Q*(∞)-*f* curves of the different probes; (**b**) *Q*(0)-*f* curves of the different probes; (**c**) *ΔQ-f* curves of the different probes.

The *Q*-factor of the coil is determined by the measured clearance, the frequency of the exacting current in the coil and the material of the measuring object. The *Q*-factors of the different probes are measured when the resonance frequency of the exciting current in the coil is varying from 50 Hz to 5 MHz. As shown in Figure 8, there are the *Q*-factors of the different probes. Figure 8(a) is the *Q*-factor when the measuring object is removed far away from the probes, which denoted as *Q*(∞). Figure 8(b) is the *Q*-factor when the measuring object is closed to the probes without any clearance which denoted as *Q*(0). Figure 8(c) is the difference of the two *Q*-factor curves listed in (a) and (b), which denoted as *ΔQ*. The linear range of the ECDS could be evaluated by the *ΔQ*.

As shown in Figure 8(c), both the *ΔQ* of the four dual-coil probes are increased compared with the single coil, but the type D dual-coil has the max *ΔQ* value, which is 12.1, much more than the 8.6 of the single-coil probe. It is means that the type D dual-coil probe potentially has a max linear range. The max value of *ΔQ* is obtained when the exciting current is 800 kHz.

## 4. The FEM Analyses of the Dual-Coil Probe

In order to evaluate the linear range extension ability of the different type dual-coil probes, the induced eddy current density and magnetic intensity distribution in the measuring object are analyzed by FEM. It is well known that, under the same conditions, the more eddy current density and magnetic intensity, the wider the linear range [14,15]. The parameters of the copper coils used in the dual-coil probe are listed in Table 1. The coils used in the single-coil probe and the dual-coil probe are identical.

**Figure 9.**
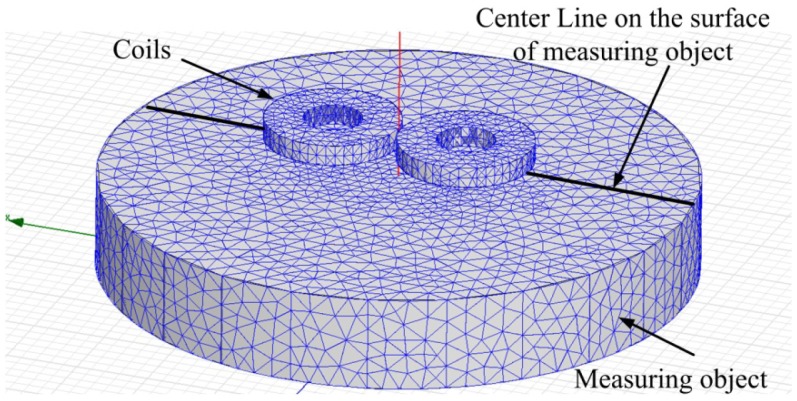
The simulation model and the mesh grids of the probe and measuring object used for FEM analysis.

As shown in Figure 9, it is the FEM model and mesh grids of the probes and the measuring object. The distance from the probe to the measuring object is 1.2 mm. It is known from the Figure 8(c) that the best exciting current frequency in the coil is 800 kHz. It is supposed that the pk-pk value of the exacting current in each coil is *I_c_* = 1 mA.

**Figure 10.**
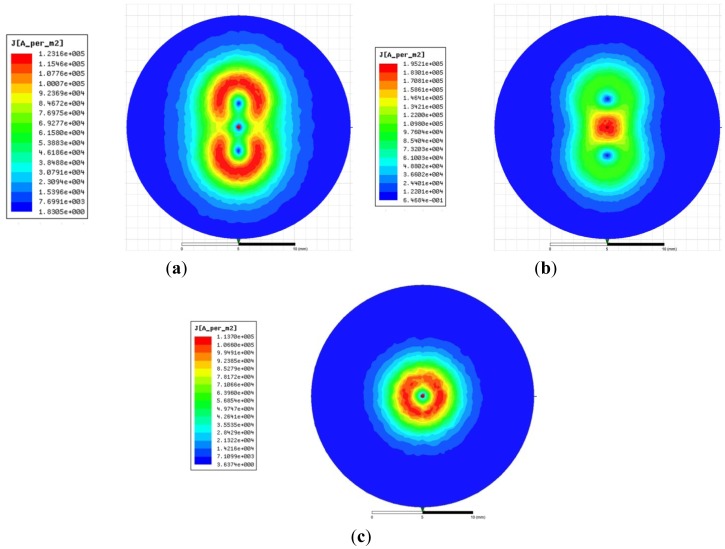
The eddy current densities on the surface of the measuring ring. (**a**) The dual-coil probe with the same direction; (**b**) The dual-coil probe with the opposite direction; (**c**) The single coil.

Because the connection modes of the two coils could not be easily separated in the FEM analysis, the exciting current direction in the two coils is the key factor taken account in the analysis. Combined with the *Q*-factor calculation results motioned above, the best type dual-coil probe could be defined. As shown in Figure 10, there are the eddy current density distributions on the surface of the measuring object while the different probes are used. The eddy current densities of dual-coil probe with same direction, dual-coil probe with opposite direction and single coil probe are 1.2316 × 10^5^ A/m^2^, 1.9521 × 10^5^ A/m^2^ and 1.137 × 10^5^ A/m^2^, respectively.

**Figure 11.**
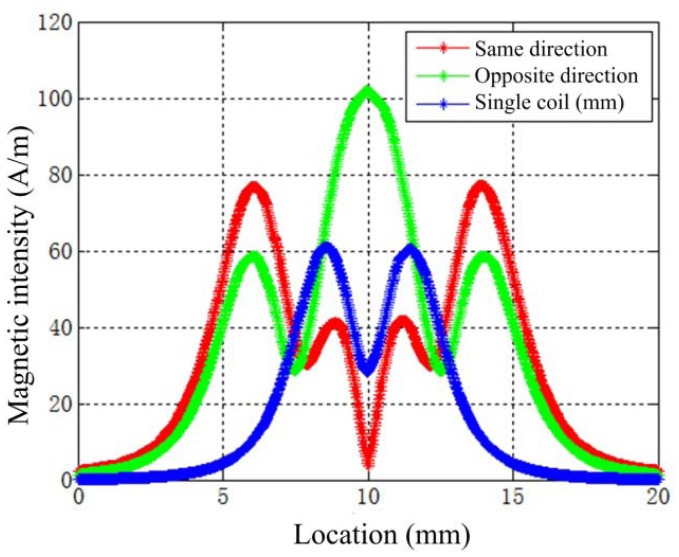
The magnetic intensity distributions along the center line on the surface of the measuring object.

Shown in Figure 11 are the magnetic intensity *H* distributions along the center line on the surface of the measuring object (described in Figure 9). The opposite connected dual-coil probes have the greatest *H* on the surface of the measuring object. The max value is 101 A/m, but it is about 60.5 A/m for a single coil probe and 78.5 A/m in the dual-coil probe with same exciting current directions.

Combining the *Q*-factor comparisons and the FEM analysis results, the type D dual-coil probe is selected as the best candidate for the wide linear range ECDS used in the G-MSFW and APH-MSFW.

## 5. Experiments and Applications

**Figure 12.**
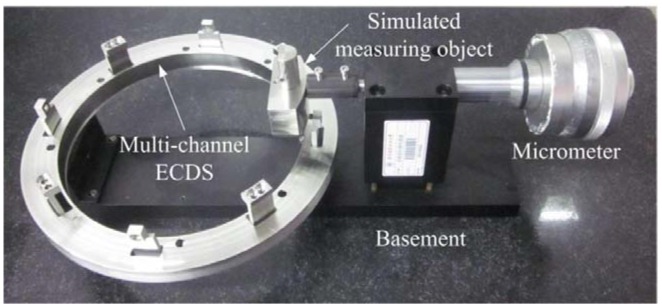
The calibration rig designed for the ECDS.

Shown in Figure 12 is the designed rig used for the calibration of the output of the ECDS. The 9-channel ECDS is mounted on the basement of the rig, the micrometer is supported by the fixed mount. A simulated measuring object made by the same metal of the actual measuring ring is interconnected to the end of the micrometer. The clearance between the probe and the simulated measuring object is driven and measured by the dial indicator of the micrometer. The voltage output of ECDS measured by the oscilloscope or voltage meter.

**Figure 13.**
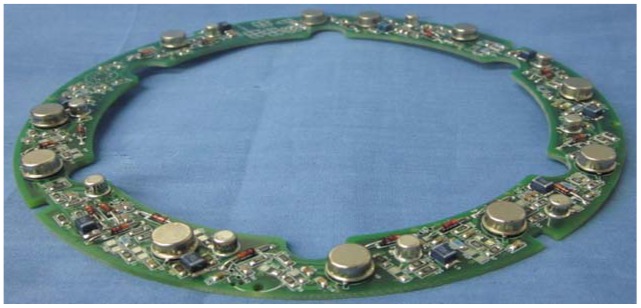
The PCB of the detecting circuits fixed in the frames of the ECDS.

The detecting circuits of all probes of the multi-channel ECDS are shown in Figure 13. It is obviously that there is not enough room for the linear compensation circuit which could extend the linear range of the ECDS easily.

**Figure 14.**
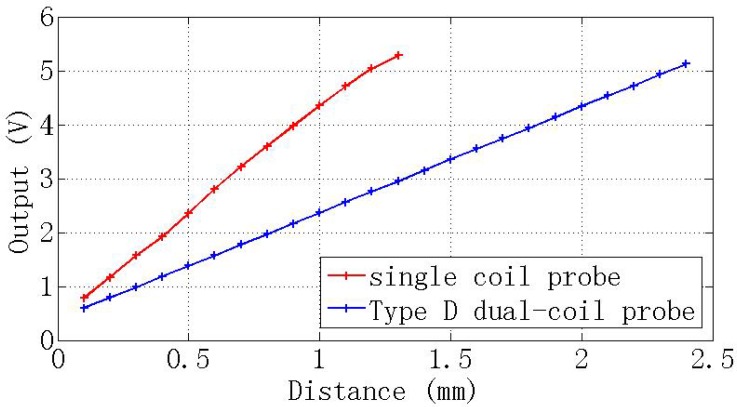
The output voltage *V*(*x*) of the ECDS.

Figure 14 shows the voltage outputs of the dual-coil ECDS equipped with the type D probe and compare with this of the ordinary single-coil ECDS. The linear range of the dual-coil ECDS is 0∼2. 4 mm, but the single-coil ECDS with the same coil parameter is 0∼1.1 mm. The linear range of the dual-coil probe ECDS is extended to more than two-times that of the traditional ECDS without any additional compensation circuit.

**Figure 15.**
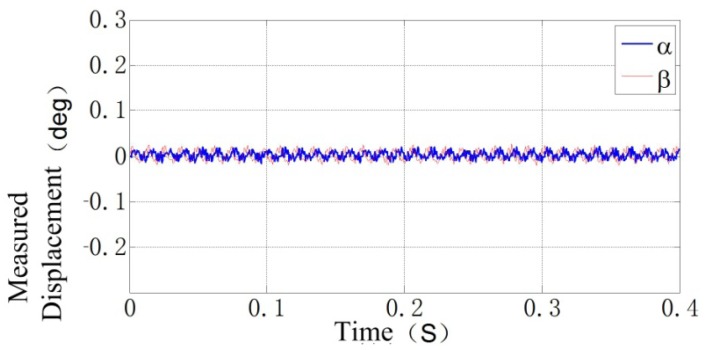
The rotor displacement locus measured by the designed dual-coil ECDS in *α* and *β* DOFs when the rotor rotates at 5,000 rpm.

Shown in Figure 15 is the rotor locus of the APH-MSFW in *α* and *β* DOFs when the rotor is rotating at 5,000 rpm. The testing results show that the dual-probe ECDS could ensure the MSFW operating in its rotating speed range ±5,000 rpm. The bandwidth of the dual-coil ECDS is more than 2.5 kHz, as same as the single-coil probe ECSP. The bandwidth is 30 times the max rotation speed of the MSFW which is 83.3 Hz (5,000 rpm), so the dynamic response of the dual-coil ECDS could satisfy the requirements of the APH-MSFW.

## 6. Conclusions

A multi-channel ECDS equipped with a dual-coil probe has been introduced in this paper to satisfy the need for wide gap measuring of the G-MSFW and APH-MSFW within the strict limitations of the coil diameter and complexity of the circuit. The *Q*-factor, eddy current density and magnetic intensity have been discussed to optimize the configuration of the two coils used in the probes. The linear range of the ECDS equipped with the best type dual-coil probe is double compared to the single-coil ECDS. The effectiveness of the linear range extension ability and the dynamic response of the new designed ECDS have been tested and confirmed by its applications in APH-MSFW. The dual-coil method mentioned in this paper is a convenient and simple method to extend the linear range of the ECDS without the cost of any additional compensation circuits or expensive coil materials.

## Figures and Tables

**Table 1.** The parameters of the coil used in the single-coil probe and the dual-coil probe.

**Parameter**	**Unit**	**Value**
The outer diameter of the coil	mm	4.7
The inner diameter of the coil	mm	2.3
The thickness of the coil	mm	0.6
The diameter of the copper wire used in the coil	mm	0.1
The turn number of each coil		66

## References

[1] Zhu K., Xiao Y., Rajendra A.U. (2009). Optimal control of the magnetic bearings for a flywheel energy storage system. Mechatronics.

[2] Sotelo G.G., De Andrade R., Ferreira A.C. (2007). Magnetic bearing sets for a flywheel system. IEEE Trans. Appl. Supercond.

[3] Boehm J., Gerber R., Kiley N. (1993). Sensors for magnetic bearings. IEEE Trans. Magn..

[4] Jansen R., Lebron R., Dever T.P., Birchenough A.G. PWM Switching Frequency Effects on Eddy Current Sensors for Magnetically Suspended Flywheel Systems.

[5] Gerlach B., Ehinger M., Raue H.K., Seiler R. Gimballing Magnetic Bearing Reaction Wheel with Digital Controller.

[6] Azukizawa T., Yamamoto S., Matsuo N. (2008). Feasibility study of a passive magnetic bearing using the ring shaped permanent magnets. IEEE Trans. Magn..

[7] Han B., Zheng S., Wang X., Yuan Q. (2012). Integral design and analysis of passive magnetic bearing and active radial magnetic bearing for agile satellite application. IEEE Trans. Magn..

[8] Yin W., Binns R., Dickinson S., Davis C., Peyton A. Analysis of the Lift-Off Effect of Phase Spectra for Eddy Current Sensors.

[9] Mizuno T., Enoki S., Hayashi T., Asahina T., Shinagawa H. (2007). Extending the linearity range of eddy-current displacement sensor with magnetoplated wire. IEEE Trans. Magn..

[10] Placko D., Dufour I. (1993). A focused-field eddy current sensor for nondestructive testing. IEEE Trans. Magn..

[11] Kim T.O., Lee G.S., Kim H.Y., Ahn J.H. (2007). Modeling of eddy current sensor using geometric and electromagnetic data. J. Mech. Sci. Technol..

[12] Sadler D.J., Ahn C.H. (2001). On-chip eddy current sensor for proximity sensing and crack detection. Sens. Actuators A: Phys..

[13] Nabavi M.R., Nihtianov S. (2011). Eddy current sensor interface for advanced industrial applications. IEEE Trans. Ind. Electron..

[14] Wilde J., Lai Y. (2003). Design optimization of an eddy current sensor using the finite-elements method. Microelectron. Reliab..

[15] Yu Y., Du P., Wang Z. Study on the Electromagnetic Properties of Eddy Current Sensor.

